# Determination of initial airtightness after anatomical laser segmentectomy in an ex vivo model

**DOI:** 10.1007/s10103-021-03312-2

**Published:** 2021-04-23

**Authors:** Andreas Kirschbaum, Andrijana Ivanovic, Thomas Wiesmann, Nikolas Mirow, Christian Meyer

**Affiliations:** 1 Department of Visceral, Thoracic and Vascular Surgery, University Hospital Gießen and Marburg (UKGM), Marburg site, Baldingerstraße, D-35043 Marburg, Germany; 2 Department of Anaesthesiology, University Hospital Gießen and Marburg (UKGM), Marburg site, Baldingerstraße, D-35043 Marburg, Germany; 3 Department of Cardiac Surgery, University Hospital Gießen and Marburg (UKGM), Marburg site, Baldingerstraße, D-35043 Marburg, Germany; 4 Department of Surgery, Asklepios Stadtklinik Bad Wildungen, Bad Wildungen, Germany

**Keywords:** Segmentectomy, Segmental resection, VATS, Laser fibre, Nd:YAG laser, Monopolar cutter, Stapler, Airtightness

## Abstract

If a pulmonary pathology can be removed by anatomical segmentectomy, the need for lobectomy is obviated. The procedure is considered oncologically equivalent and saves healthy lung tissue. In every segmentectomy, lung parenchyma must be transected in the intersegmental plane. Using an ex vivo model based on porcine lung, three transection techniques (monopolar cutter + suture, stapler, and Nd:YAG laser) are to be compared with respect to their initial airtightness. At an inspiratory ventilation pressure of 25 mbar, all three preparations were airtight. Upon further increase in ventilation pressure up to 40 mbar, the laser group performed best in terms of airtightness. Since thanks to its use of a laser fibre, this technique is particularly suitable for minimally invasive surgery; it should be further evaluated clinically for this indication in the future.

## Background

If a pulmonary focal lesion is limited to one segment, it is removed by anatomical segmentectomy, in particular if a malignancy is suspected. Only if the lymph nodes near the outlet of the segmental bronchus are tumour-infiltrated, should lobectomy be performed. Despite the parenchyma-saving nature of the procedure, several studies have shown that patients who underwent segmentectomy had the same long-term survival in lung cancer as those who underwent lobectomy had [1–3]. Anatomical segmentectomy can be performed either by open or by minimally invasive surgery. After exposure of the pulmonary artery segment branch, the same is transected. The segmental bronchus is then exposed to the extent that it can be clamped out. After inflation of the lung, the associated atelectatic segment demarcates itself. Once the lung is deflated again, the segmental bronchus is occluded using a stapling device and transected. The segment is then removed by severing a parenchymatous bridge to the remaining lung. There are various technical options available for this surgical step. The parenchymatous bridge can be cut step by step with a monopolar cutter [4]. Punctual bleeding can be coagulated locally. In addition, the resection area is continuously sealed overhand with a monofilament thread [5]. This is intended to reduce air leakage when the lungs are re-inflated. This procedure is time-consuming and problematic in the case of pre-damaged lung tissue, as the sutures find little support when the lung is re-inflated, which can lead to considerable air leakage.

In many centres, a stapling device [6, 7] is used for occlusion and removal of the segment. The device consists of a handle and a magazine section. The magazine section can be replaced after use. Magazines of various lengths and staple heights are used. In its application, the lung parenchyma is grasped with the instrument branches. After the closing of the instrument branches, the lung parenchyma is compressed. After actuation on the instrument handle, three-row clamps are placed. The lung parenchyma is thus sealed. Next, the lung parenchyma is transected with a knife integrated into the stapler. Seeping haemorrhages from the staple rows occur rarely. These haemorrhages are usually controlled by compression; sutures are necessary in fewer cases. In most cases, several magazines are required to cut through the lung parenchyma bridge in the course of a segmentectomy, which makes such a surgery expensive. If the lung parenchyma to be transected is wide, the staples may be torn off after re-inflation of the lung, with subsequent air leakage. This area must then be closed with a suture. In most cases, a collagen fleece is additionally glued on. All these efforts are intended to avoid persistent air loss in the postoperative course. These necessitate a longer indwelling time of the thoracic drainage and thus lead to longer hospitalisation.

In many hospitals, pulmonary metastases are removed in a parenchyma-saving way using an Nd:YAG laser (wavelength 1320 nm) [8, 9]. Here, the laser light incides onto the lung parenchyma. After absorption of the light in the tissue, it is converted into thermal energy. Temperatures far above 1000 °C are generated, which lead to local vaporisation of the tissue. The temperature declines towards the periphery, so the tissue is only coagulated there. This allows for a procedure with low bleeding rates. In previous studies [10, 11], we were able to show that down to a resection depth of 1.5 cm, there was sufficient airtightness, so that the resection area did not need to be sealed with a suture.

Using an ex vivo model based on porcine lung, we want to investigate whether there is sufficient initial airtightness after anatomical laser segmentectomy to obviate the need for suturing of the coagulated resection area. These results are to be compared and discussed with those of the other two techniques above.

## Materials and methods

**Fig 1:**
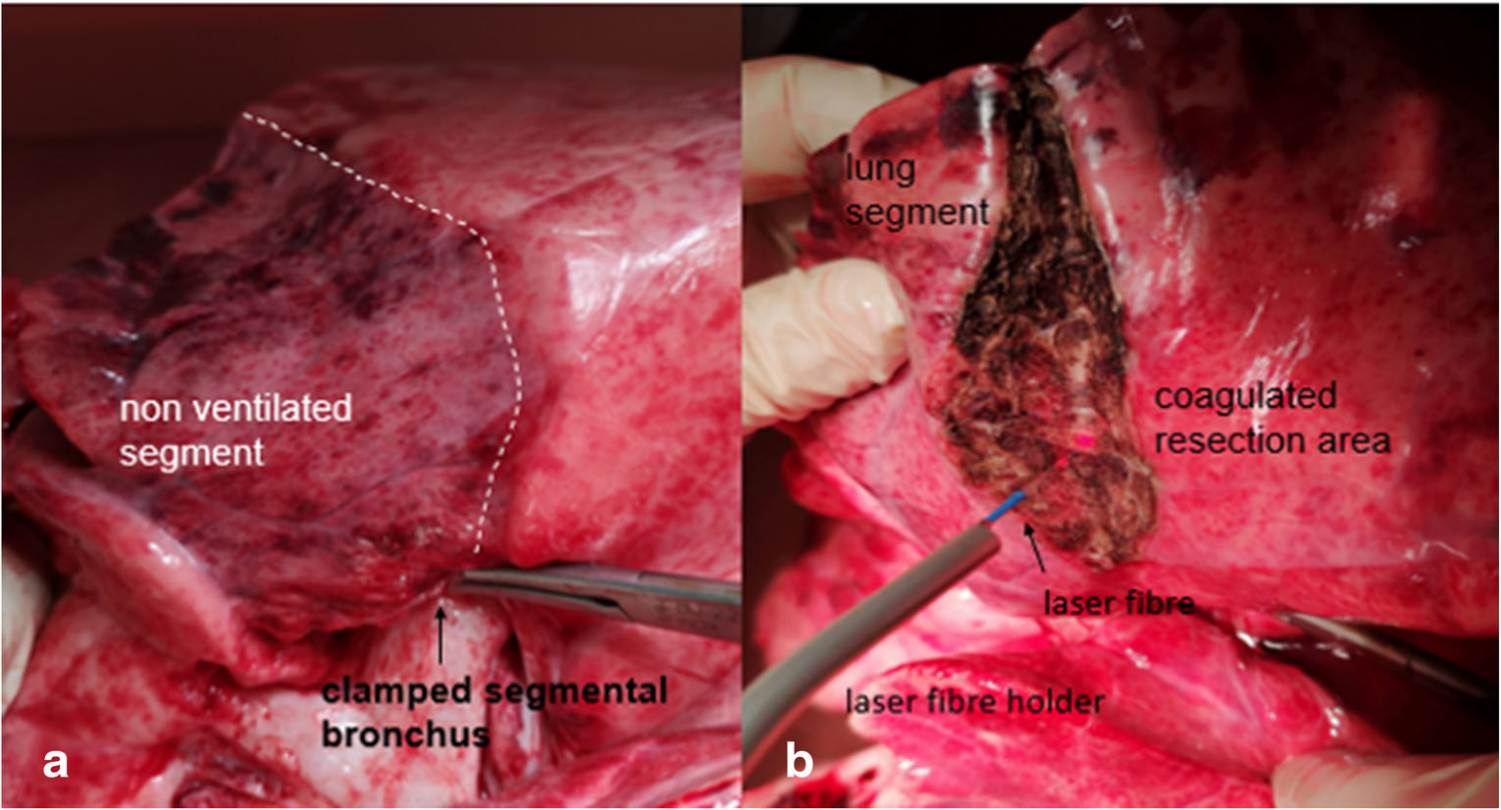
**a** Marked atelectatic segment of the lower lobe after clamping out of the segmental bronchus. **b** Segmentectomy through the use of a laser fibre (800 μm). The laser fibre is handled with a fibre holder (own photo)

The heart and both lungs of freshly slaughtered pigs (EU standard: 90 kg) were removed as an organ package. For this purpose, the proximal trachea and the oesophagus were severed and thus both organs removed. The specimens were inspected for their integrity; only those that were completely undamaged were used for the experiments. After the packaging of the preparations, these were immediately transported to our laboratory. In the laboratory, the oesophagus and the remains of the aorta and pericardium were removed from each preparation. After identification of the inter-lobar gap, the lobar bronchi were identified, and the respective segmental bronchi were identified by exposure. Next, the trachea was intubated with a ventilation tube (Vygon 520, CH 8.0, Braun Melsungen, Germany), and the latter was blocked. All secretion from the tube was aspirated. The segmental bronchus was then clamped out with a clamp (Fig. 1a). The lungs were aerated with pressure-controlled ventilation (Cicero EM, PM8060, Dräger Lübeck, Germany, frequency 10/min, *p*_insp_ = 20 mbar, PEEP = +5 mbar). The atelectatic segment was identified, and its boundaries were clearly delineated with a marker pen. Ventilation was stopped, causing the entire lung to collapse. Depending on the technique to be used, three groups (*n* = 12 each) were formed. The allocation of the preparations to the individual groups was random. In group 1, the lung parenchyma was severed using a monopolar cutter. A sealing suture was then provided using a continuous monofilament (PDS 2, USP 4-0, Johnson & Johnson, Norderstedt, Germany). In group 2, the lung parenchyma was occluded and severed by several strokes of a stapler (Tri-Staple™, Medtronic, Minneapolis, USA). In group 3, a laser fibre (800 μm) connected to an Nd:YAG laser (wavelength 1320 nm, laser power: 60 W) (Gebrüder Martin & Co. KG, Tuttlingen, Germany) was used. The laser fibre was handled with a fibre holder as used in video-assisted surgery (Fig 1b). Care was always taken during use of the laser fibre to ensure that it did not show any signs of melting of the fibre end. Whenever this occurred, the fibre was immediately prepared freshly. If small bronchial tubes were clearly opened after the transection of the lung parenchyma, they were sutured up with a monofilament thread (PDS2, USP 4-0, Johnson & Johnson, Norderstedt, Germany). At the end of the parenchyma transection, the resection areas were additionally coagulated using the laser.

**Fig 2:**
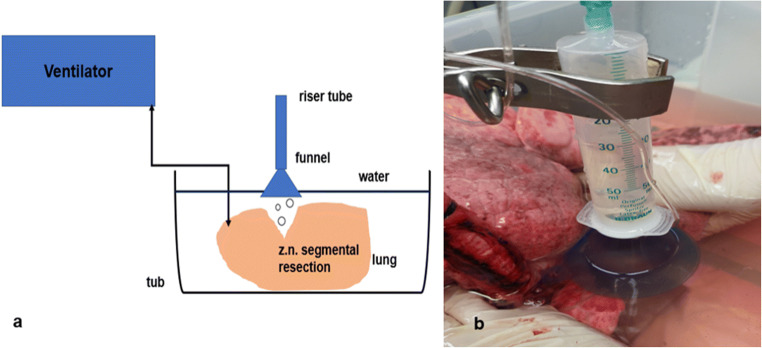
**a** Illustration of the experimental setup. **b** Quantitative determination of the air leakage by collecting the escaping air through a funnel (own photo)

After completion of the respective parenchyma transection, a plexiglass tub was filled with water, and ventilation was resumed (Fig. 2a). The airtightness of the respective parenchyma resection area was assessed at an inspiratory ventilation pressure of 25 mbar. This pressure corresponds to that which we apply intra-operatively for the leakage test [12]. An established score [13] was used for this purpose: Score 0 = completely airtight, score 1 = escape of individual bubbles, score 2 = emission of a chain of bubbles, and score 3= massive air outflow. The location of the air leak was noted. To better quantify the amount of air escaped per ventilation, a funnel connected to a riser pipe was positioned over the leak. The riser pipe was filled with water up to a mark. The rising air displaces the water. The markings on the riser tube showed the respective air volume in ml per ventilation stroke (Fig. 2b). If there was no air leakage at an inspiratory pressure of 25 mbar, the latter was increased in steps of 5 mbar to a maximum of 40 mbar. Five respiratory cycles were performed at each pressure stage before the ventilation pressure was increased again.

The proportion of airtight resection areas per group was evaluated at an inspiratory pressure of 25 mbar, as well as at a maximum inspiratory pressure of 40 mbar.

## Results

### Airtightness of the resection area at *p*_ins_ = 25 mbar

In all groups, the resection area was completely airtight at an inspiratory ventilation pressure of 25 mbar. The usual intra-operative control of the lung parenchyma for airtightness does not show any differences between the individual methods.

### Airtightness of the resection area at a maximum *p*_insp_ = 40 mbar

In group 1, two resection areas were leaky at a ventilation pressure of 30 mbar. One I° and one II° leakage occurred. Ten resection areas (83.3%) were completely airtight at this pressure. When the ventilation pressure was increased to 35 mbar, one resection area additionally showed a II° leakage. Nine resection areas (75%) were still airtight. At a maximum ventilation pressure of 40 mbar, 7 sutures (58.3%) were airtight, while two others became leaky. One I° and one II° leakage occurred. In group 2, 11 resection areas (91.6%) were airtight at a ventilation pressure of 30 mbar. Only one staple seam showed a II° leakage. At a ventilation pressure of 35 mbar, three staple seams became leaky. This resulted in two II° leakages and one III°. Eight staple seams (66.6%) were completely airtight at this pressure. At the maximum ventilation pressure of 40 mbar, three further staple seams became leaky. The leakages were one I°, one II°, and one III°. Five staple seams (41.6%) were still airtight at this maximum pressure.

In the laser group, all resection areas (100%) were completely airtight at a ventilation pressure of 30 mbar. When the ventilation pressure was increased to 35 mbar, two resection areas became punctually leaky. One I° and one III° leakage occurred. Upon closer inspection of the resection area, small segmental bronchi were open, which were the cause of the leakage. Ten resection areas (83.3%) were completely airtight at a ventilation pressure of 35 mbar. At a maximum ventilation pressure of 40 mbar, two additional resection areas were leaking. One II° and one III° leakage occurred. In these cases, too, the air leaks could be explained by severed sub-segmental bronchi. The actual coagulated lung parenchyma, by contrast, was completely airtight. Eight resection areas (66.6%) were completely airtight even at this high ventilation pressure.

**Fig. 3:**
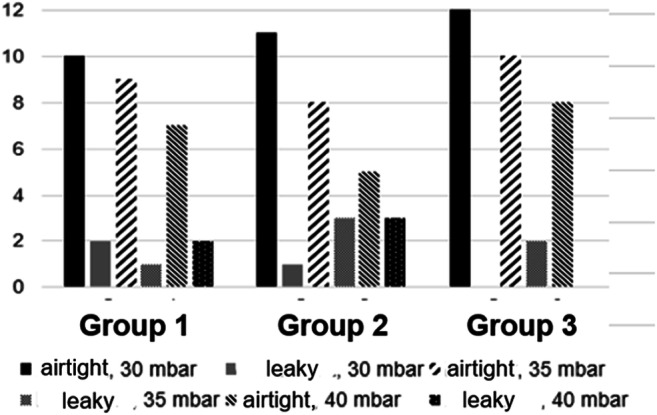
Airtightness of the resection areas in groups 1 to 3, graded up to a maximum *p*_ins_ = 40 mbar

**Fig. 4:**
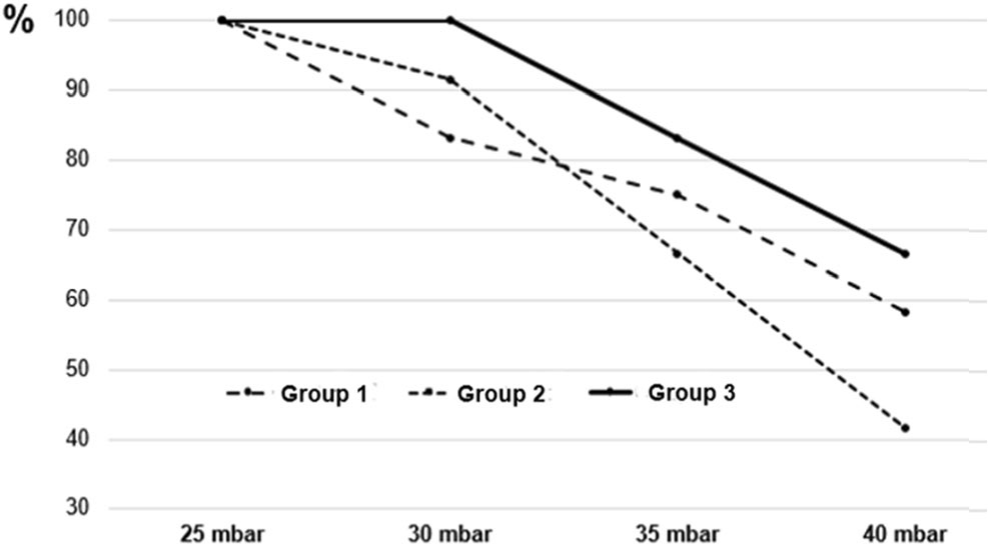
Relative airtightness of groups 1 to 3 with increasing inspiratory ventilation pressure

Table 1 and Figs. 3 and 4 show an overview of the results.

**Table 1:** Leakiness of the resection surfaces at a maximum inspiratory pressure of 40 mbar, groups 1–3 in comparison

	Airtight	Leaky	Score	Air volume escaped (ml/min)
Group 1				
30 mbar	10	2	1 × I 1 × II	1.0 8.0
35 mbar	9	1	1 × II	1.0
40 mbar	7	2	1 × I 1 × II	1.0 1.0
Group 2				
30 mbar	11	1	1 × II	1.0
35 mbar	8	3	2 × II 1 × III	1.0 14.0
40 mbar	5	3	1 × I 1 × II 1 × III	1.0 1.0 8.0
Group 3				
30 mbar	12	0	0	0
35 mbar	10	2	1 × I 1 × III	1.0 6.0
40 mbar	8	2	1 × II 1 × III	2.0 16.0

## Discussion

If a pathology is limited to a pulmonary segment, it can be removed by anatomical segmentectomy. Even in the case of a malignant focal lesion, lobectomy and thus loss of healthy lung parenchyma can be obviated. In every segmentectomy, after transection of the incoming bronchi and vessels, a parenchymatous bridge must be cut. In case of failure to adhere exactly to the segment border, air leaks may occur in the further postoperative course. These are often the cause of prolonged hospitalisations. In the present work, we have used an ex vivo model of the porcine lung to study three techniques of parenchyma transection with respect to initial airtightness. In the first technique, the lung parenchyma was transected at the segment border using a monopolar cutter. Afterwards, the edges of the resection area were continuously sealed with an overhand suture. In the second technique, the lung parenchyma was occluded and severed using a stapling device. In the last technique, the lung parenchyma was cut using a laser fibre connected to an Nd:YAG laser. The resection area was additionally coagulated over the entire surface and not sealed with a suture.

All the techniques showed no air leakage at an inspiratory pressure of 25 mbar. Since this pressure is the intra-operative test pressure for airtightness [12], all three techniques can in principle be used for parenchyma transection in the course of segmentectomy. Differences occurred only when the ventilation pressures were further increased to 40 mbar. It was found that the staple group had much lower resistance to the pressure load. This aspect is surprising because in a study by Chen et al. [14], the stapler was superior to the monopolar cutter. A conceivable cause could be that the staples tend to loosen and produce leakage if the parenchymatous bridge is wider. The first group also showed increased air leakage upon rising pressure. After all, at an inspiratory pressure of 40 mbar, 7 preparations (58.3%) were still airtight. As a rule, the seams became leaky by cutting into the fabric and tearing out. This effect could be further enhanced in structurally altered lung tissue, e.g., in pulmonary emphysema. These results were confirmed by a study by Liu et al. [15].

The laser group achieved the best results. At a maximum pressure of 40 mbar, 8 preparations (66.6%) were airtight, and two preparations were leaking for the first time. It is important here to make the cut exactly in the intersegmental plane. If this is not successful, the risk of opening larger segmental bronchi is significantly increased [10, 11]. The leakages in the laser group were exclusively caused by the punctual opening of small bronchial tubes. Therefore, the resection surface should always be inspected for such areas. In this situation, we recommend occluding these small bronchial tubes with a suture or clip. It was noticeable that the rest of the lung parenchyma was completely airtight. We attribute this to the coagulation of the surface. The technique with a laser fibre clamped into a fibre holder is particularly suitable for minimally invasive surgery. Under visual control, the lung parenchyma can be severed step by step. This process can be supported by manual tension on the lung parenchyma. This allows the work to be carried out quickly. To achieve an optimal effect of the laser fibre on the tissue, care should be taken to ensure that the fibre tip is not melted. If this has occurred, the fibre tip must be freshly prepared.

The ex vivo model which we have created specifically for this investigation is particularly suitable for the standardised and reproducible study of the process of parenchyma transection in the course of anatomical segmentectomy. Detection of the segment to be removed succeeded in each case without any problems by clamping out the segmental bronchus. Since we used preparations from healthy pigs only, our examination refers to normal lung tissue. It is possible that differing results were to be expected with structurally altered lung tissue. In our investigation, we focused on the initial airtightness at a ventilation pressure of 25 mbar, which is the standard ventilation pressure in hospitals [12]. Unfortunately, it is not possible to conduct a long-term study with the model used. It would be interesting to know whether after a certain period of time, the leakage rate has changed in relation to the individual techniques.

Our results indicate that the laser can be rationally applied in the context of anatomical segmentectomy. Particularly in minimally invasive procedures, it may be advantageous in order to shorten the operating time, as the resection area no longer needs to be sealed with sutures. Compared to the other two techniques, the laser tends to perform better. The next step should now be an in vivo study in pigs to further investigate the place of laser application in the context of minimally invasive anatomical segmentectomy. If the results are positive, a randomised clinical study should be considered to explore the use of a laser in the course of minimally invasive anatomical segmentectomy in comparison to the conventional stapler application.

## Conclusion

A parenchymatous bridge must be transected in any anatomical segmentectomy. To avoid complications, the cut should be made exactly in the intersegmental plane. Compared to the established techniques, use of an Nd:YAG laser does not yield worse results in this indication. This technique is suitable in particular for minimally invasive use and should be evaluated further.
